# Attentional Orienting and Dorsal Visual Stream Decline: Review of Behavioral and EEG Studies

**DOI:** 10.3389/fnagi.2017.00246

**Published:** 2017-07-27

**Authors:** Evatte T. Sciberras-Lim, Anthony J. Lambert

**Affiliations:** Department of Psychology, University of Auckland Auckland, New Zealand

**Keywords:** aging, visuospatial orienting of attention, compensation, dorsal visual stream, magnocellular pathway

## Abstract

Every day we are faced with an overwhelming influx of visual information. Visual attention acts as the filtering mechanism that enables us to focus our limited neural resources, by selectively processing only the most relevant and/or salient aspects of our visual environment. The ability to shift attention to the most behaviorally relevant items enables us to successfully navigate and interact with our surroundings. The dorsal visual stream is important for the rapid and efficient visuospatial orienting of attention. Unfortunately, recent evidence suggests that the dorsal visual stream may be especially vulnerable to age-related decline, with significant deterioration becoming evident quite early in the aging process. Yet, despite the significant age-related declines to the dorsal visual stream, the visuospatial orienting of attention appears relatively well preserved in older adults, at least in the early stages of aging. The maintenance of visuospatial orienting of attention in older adults appears to be facilitated by the engagement of compensatory neural mechanisms. In particular, older adults demonstrate heightened activity in the frontal regions to compensate for the reduced activity in the posterior sensory regions. These findings suggest that older adults are more reliant on control processes mediated by the anterior regions of the frontoparietal attention network to compensate for less efficient sensory processing within the posterior sensory cortices.

## Introduction

Everything from playing tennis to simply walking down the street require the successful orienting of attention from one location to the next. This process of shifting attention is referred to as the visuospatial orienting of attention. A process which relies on an interacting network of structures, which include regions of the lateral prefrontal cortex (Corbetta and Shulman, 2002, 2011; Corbetta et al., 2008; Schall, 2009; Vandenberghe and Gillebert, 2009; Asplund et al., 2010), working in conjunction with the dorsal visual stream (Siegel et al., 2008; Lambert and Shin, 2010; Marrett et al., 2011; Capilla et al., 2014) to guide the deployment of attentional resources. The involvement of dorsal visual stream processing in visuospatial orienting is consistent with both Goodale and Milner’s (1992) duplex model of vision which suggests that the dorsal visual stream is responsible for visually guided actions (including eye movements; Milner and Goodale, 2008), and with the premotor theory of attention which suggests that there is considerable overlap in the neural mechanisms responsible for the overt and covert orienting of attention (Rizzolatti et al., 1987). Unfortunately, recent evidence suggests that the dorsal visual stream may be vulnerable to age-related declines from relatively early on in the aging process (Langrová et al., 2006). This mini-review article examines the impact of these age-related declines on the visuospatial orienting of attention.

## Age-Related Decline of The Dorsal Visual Stream

Recent studies suggest that the dorsal visual stream may be vulnerable to age-related atrophy and decline due to its cellular composition. The dorsal visual stream which extends from area V1 to the dorsolateral occipital cortex (Maunsell, 1987), and regions of the posterior parietal cortex (Kravitz et al., 2011), receives input primarily from the magnocellular layers of the lateral geniculate nucleus (LGN; Celesia and DeMarco, 1994; Grill-Spector and Malach, 2004). In contrast, the ventral visual stream which extends from area V1 to the inferior temporal cortex (Kravitz et al., 2011), receives input from both the magnocelluar and parvocellular layers (Celesia and DeMarco, 1994; Grill-Spector and Malach, 2004) of the LGN. Braddick et al.’s (2003) dorsal stream vulnerability hypothesis argues that, since magno cells are larger than parvo cells, and neurons with larger cell bodies and axon diameters are more susceptible to damage, magno cells are more susceptible to degeneration. In addition, losses in the magnocellular pathway may be more readily apparent as there are far fewer magno cells than parvo cells (Skottun, 2000). Thus, even when similar numbers of neurons are lost in both the magnocellular and parvocellular pathways, functional declines may be more apparent in the magnocellular pathway. Since the dorsal visual stream’s primary input comes from magno cells (Celesia and DeMarco, 1994), the dorsal visual stream is in turn, more susceptible to degeneration (Braddick and Atkinson, 2011). Although Braddick’s proposal of dorsal visual stream vulnerability was based on an examination of dorsal visual stream deficits in early childhood development; it does highlight the possibility that the dorsal visual stream may be more susceptible to decline in general.

Evidence of age-related dorsal visual stream declines has come from studies examining the age-related declines to neural structures. In a study by Ziegler et al. (2012), 547 participants between the ages of 19–86 years old were recruited, to perform a large-sample cross-sectional voxel-based morphometry (VBM) study to examine reductions in gray matter volume with advancing age. The results revealed that the dorsal visual stream exhibited substantially larger reductions in gray matter volume, compared to the ventral visual stream and the primary visual areas. These declines have been localized to the superior parietal cortex (Driscoll et al., 2009), as well as, the superior and inferior parietal gyri (Crivello et al., 2014; Fjell et al., 2014), all of which are crucial for the visuospatial orienting of attention (Corbetta and Shulman, 2002). Moreover, in elderly subjects, neural declines in the parietal regions (Resnick et al., 2003; Driscoll et al., 2009; Thambisetty et al., 2010; Crivello et al., 2014; Fjell et al., 2014) are compounded by a reduction in cerebral blood flow to the region (Martin et al., 1991), resulting in less efficient processing within the dorsal visual stream.

## Impact of Dorsal Visual Stream Declines (Behavioral)

This mini-review article will focus primarily on the impact of these declines on the age-related changes to performance within the spatial cueing paradigm. In these studies, a cue elicits a shift of attention to its location (in the case of a peripheral cue; Eriksen and Hoffman, 1974; Theeuwes, 1991; Rafal and Henik, 1994; Yantis and Hillstrom, 1994; Oonk and Abrams, 1998), or to the location signaled by the cue stimulus (in the case of a central cue; Jonides, 1981; Shepherd and Müller, 1989; Cheal and Lyon, 1991; Theeuwes, 1991; Friesen et al., 2004). Subsequently, a target appears in either the location signaled by the cue stimulus (valid trial), or at an un-cued location (invalid trial). Participants are tasked with making a response to the onset of the target stimulus. The cueing benefit is indexed by a decrease in reaction times when targets appear in the location signaled by the cue stimulus and is believed to reflect the engagement of attentional resources at the target location (e.g., Jonides, 1981; Shepherd and Müller, 1989; Theeuwes, 1991; Yantis and Hillstrom, 1994; Friesen et al., 2004). Conversely, the cueing cost is indexed by an increase in reaction times when the target appears at an un-cued location, and is believed to reflect the disengagement and shifting of attentional resources from the invalidly cued location to the target location (Posner, 1980).

It appears that despite the declines to the dorsal visual stream, the visuospatial orienting of attention appears relatively well-preserved in older adults (Nissen and Corkin, 1985; Hartley et al., 1990). In spite of the slower reaction times seen in older adults, the magnitude of cueing benefit for both peripherally (Folk and Hoyer, 1992, Experiment 1; Greenwood et al., 1993; Olk and Kingstone, 2009) and centrally (Greenwood et al., 1993; Lincourt et al., 1997; Curran et al., 2001; Lorenzo-López et al., 2002; Olk and Kingstone, 2009) presented cues is similar for both younger and older adults. However, some studies have noted an increase in cueing costs following invalidly cued targets (Hartley et al., 1990; Greenwood and Parasuraman, 1994). Taken together, these results suggest that aging may be selectively associated with reduced efficiency in disengaging and shifting attentional resources from one location to the next, while the ability to effectively utilize the cues to guide the deployment of attentional resources remains intact.

One potential shortcoming of the aforementioned studies is that in these studies participants are required to make a detection or discrimination response to a target in an otherwise empty screen. In contrast, visual scenes are typically cluttered and we need to be able to rapidly identify a relevant item (target) from surrounding items (distractors). A process which has typically been studied using the visual search paradigm (Treisman and Gelade, 1980). In these studies, participants are required to identify a target that differs from surrounding items by a single feature (feature search) or by a conjunction of features (conjunction search; Treisman and Gelade, 1980). Results indicate that aging appears to selectively impair conjunction search while feature search remains intact (Plude and Doussard-Roosevelt, 1989). An elegant study by Greenwood and Parasuraman (1999) examined if the selective age-related impairment in conjunction search could in part be accounted for by an age-related reduction in the ability to flexibly expand and contract the focus of attention. To do so, they employed the use of precues that indicated the location of an upcoming target with a varying degree of precision (element-size precue: highlights a single possible location; column-size precue: highlights a column of possible locations; array-size precue: highlights a whole array of potential locations). The results indicated that age-related impairments in conjunction search can be alleviated by the use of precise and valid precues. Although this benefit was somewhat smaller in the oldest group of participants (above 76 years old). They suggest that aging impairs the ability to flexibly expand and contract the focus of attention and that elderly participants are more reliant on the precues to adjust the scale of their attention. But in the oldest participants the ability to utilize the precues to adjust the scale of attention is reduced. These findings suggest that although the visuospatial orienting of attention remains relatively well preserved in older adults, its flexibility is somewhat reduced.

## Impact of Dorsal Visual Stream Declines (EEG)

Additionally, some studies have also employed the use of electroencephalographic (EEG) recordings in order to examine the electrophysiological correlates of attention shifts. These event-related potential (ERP) studies have most commonly focused on the P1 (80–130 ms) and the N1 (140–200 ms) components in relation to processing of target stimuli (Mangun and Hillyard, 1991; Wright et al., 1995). These studies demonstrate that shifting attention to a particular location of the visual field, increases the amplitude of P1 and N1 components (Mangun and Hillyard, 1991; Eimer, 1994; Luck et al., 1994; Mangun, 1995; Anllo-Vento et al., 1998) evoked by stimuli within the attended location. The amplification of these components appears to involve a selective enhancement of the signal to noise ratio of stimuli within the attended area, thereby strengthening the perceptual representation of stimuli located within that region (see Carrasco, 2011, for review; Heinze et al., 1990, 1994). Consequently, stimuli within the attended area are more rapidly detected; giving rise to more rapid response times when targets appear at the cued location (valid trials) compared to when targets appear at un-cued locations (invalid trials). Results from ERP studies examining the age-related changes to the underlying neural substrates of visuospatial orienting closely parallel the results of behavioral studies. These studies demonstrate that despite delayed latency of the P1 (80–130 ms) and N1 (140–200 ms) components, the augmentation of these early components elicited by attended relative to unattended targets is similar across both younger and older adults (Yamaguchi et al., 1995; Curran et al., 2001; Nagamatsu et al., 2011). With some studies showing linear correlations between the latency of ERP components and the mean reaction times of elderly participants (Li et al., 2013), which further bolsters the proposal that reductions in transmission efficiency of neural signals may account for slowed visuospatial orienting in elderly subjects (Hong and Rebec, 2012).

More recently, researchers have begun to focus on the neural activity elicited during the cue-target interval; much of this work has focused upon examining the modulation of prestimulus alpha activity along the fronto-occipital axis during the cue-target interval (Foxe et al., 1998; Worden et al., 2000; Babiloni et al., 2006; Rihs et al., 2009; Foxe and Snyder, 2011). Alpha-band desynchronization of the contralateral fronto-occipital axis appears to increase perceptual sensitivity by causing a baseline shift in the sensitivity of the neurons representing the to-be-attended location (Sauseng et al., 2005; Rihs et al., 2007; Siegel et al., 2008; Capotosto et al., 2009; Kelly et al., 2009; Capilla et al., 2014), while a concurrent alpha-band synchronization of the ipsilateral fronto-occipital axis inhibits processing of unattended regions (Kelly et al., 2006; Klimesch et al., 2007; Rihs et al., 2007, 2009; Capotosto et al., 2009; Foxe and Snyder, 2011; Bengson et al., 2012). The lateralization of prestimulus alpha reflects the top-down attentional modulation of neural processing in the visual cortices, and is referred to as proactive attentional control (Braver, 2012). It is termed proactive as it reflects the ability of the attentional system to bias perceptual processing in favor of an upcoming target before it is presented. In contrast, reactive attentional control refers to resolving interference between target and potentially distracting information at later stages of the processing hierarchy (Geerligs et al., 2014).

Recent studies indicate that the level of alpha power lateralization during the cue-target interval is significantly reduced in older adults (Vaden et al., 2012; Hong et al., 2015; Li and Zhao, 2015), and this reduction is most prominent along parietal-occipital regions (Zanto et al., 2011; Deiber et al., 2013). Specifically, older adults showed significant reductions in the level of event-related synchronization of prestimulus alpha (ipsilaterally) along these sites (Karrasch et al., 2004; Deiber et al., 2010; Vaden et al., 2012), which is consistent with earlier reports suggesting that older adults face significant difficulty with distractor suppression (Gazzaley et al., 2008; Schmitz et al., 2010; Haring et al., 2013). The decreased modulation of prestimulus alpha is proposed to be the result of the overall decline in alpha power in older adults, which renders the modulation of prestimulus alpha a less efficient means of attentional control (Vaden et al., 2012; Deiber et al., 2013; Hong et al., 2015; Li and Zhao, 2015). Older adults may compensate for this deficit with stronger early engagement of motor areas (Deiber et al., 2013), and an increase in reactive control (Paxton et al., 2008; Geerligs et al., 2014) mediated by the anterior nodes of the frontoparietal attention network (De Fockert et al., 2009; Schmitz et al., 2010; Haring et al., 2013; Li et al., 2013; Geerligs et al., 2014). These findings suggest that the age-related deterioration of parietal-occipital regions impairs elderly participants’ ability to engage in the proactive attentional biasing of early sensory regions and leads to an increased reliance on more reactive control strategies.

Additionally, while the majority of research into the patterns of age-related cerebral reorganization have centered on intra-hemispheric patterns of reorganization, there is also extensive evidence to support inter-hemispheric patterns of reorganization (Cabeza et al., 1997; Madden et al., 1999; Tulving et al., 1994; Reuter-Lorenz et al., 2000; Cappell et al., 2010). It has been proposed that with the progressive age-related deterioration of specialized neural networks, the high metabolic costs of inter-hemispheric communication (Bullmore and Sporns, 2012; Liang et al., 2013) are outweighed by the benefits to behavioral performance (Banich, 1998; Cabeza, 2002). Although most studies of age-related asymmetry reduction have focused predominantly on the bilateral recruitment of the prefrontal cortices (Madden et al., 1999; Reuter-Lorenz et al., 2000), there is also evidence for age-related asymmetry reduction within the parietal cortices (Garavan et al., 1999). This implies that while the control of spatial attention may be strongly right lateralized in young adults (Corbetta et al., 1993; Foxe et al., 2003; Thiebaut de Schotten et al., 2011), older adults may maintain their performance on visuospatial orienting tasks by the bilateral recruitment of the posterior parietal cortex. Evidence for the hemispheric asymmetry reduction in visuospatial attention comes from studies demonstrating age-related attenuation of pseudoneglect in healthy older adults (Schmitz and Peigneux, 2011; Benwell et al., 2014; Learmonth et al., 2017). Pseudoneglect refers to the consistent attentional bias to the left visual field that is typically observed in healthy young adults (Bowers and Heilman, 1980; Voyer et al., 2012), and is believed to be due to the right hemisphere dominance for visuospatial processing (Waberski et al., 2008; Cavézian et al., 2012). Learmonth et al. (2017) demonstrated the typical leftward attentional bias in young adults was coupled with greater activity over the right parieto-occipital regions, and this lateralization was absent in older adults (whom also failed to show a leftward attentional bias). These results suggest that in older adults’ deterioration of parieto-occipital regions may lead to compensatory increases in activity from homologous regions within the opposite hemisphere.

## Conclusion

In summary, these studies suggest that in spite of the early age-related declines of the dorsal visual stream the visuospatial orienting of attention remains relatively well preserved, at least in the earlier stages of aging. This maintenance appears to be facilitated by the engagement of compensatory neural mechanisms; which is consistent with both the compensation-related utilization of neural circuits hypothesis (CRUNCH; Reuter-Lorenz and Cappell, 2008), and the scaffolding theory of aging and cognition (STAC; Park and Reuter-Lorenz, 2009), which propose that the age-related deterioration of the frontoparietal attention network will result in the engagement of compensatory neural mechanisms to support the performance of visual attention tasks. Although the engagement of compensatory neural mechanisms enables older adults to shift attention in the face of widespread structural deterioration (Schneider-Garces et al., 2010; Vallesi et al., 2011; Shafto et al., 2012; Meunier et al., 2014), it results in slower and less efficient task performance (Park and Reuter-Lorenz, 2009; Meunier et al., 2014; Reuter-Lorenz and Park, 2014). Furthermore, progressive deterioration of the frontoparietal network will result in an increased reliance on compensatory neural mechanisms, but at the same time increasing levels of atrophy and structural deterioration also limits the brain’s capacity for reorganization (Burke and Barnes, 2006). As age-related atrophy proceeds, the brain will eventually reach its limits for functional reorganization and result in the more apparent attentional declines seen in the later stages of old age (80 years and above; Daffner et al., 2011).

## Author Contributions

The mini review was written by ETS-L with assistance and feedback from AJL.

## Conflict of Interest Statement

The authors declare that the research was conducted in the absence of any commercial or financial relationships that could be construed as a potential conflict of interest.
